# High-density genetic map construction and QTLs analysis of grain yield-related traits in Sesame (*Sesamum indicum* L.) based on RAD-Seq techonology

**DOI:** 10.1186/s12870-014-0274-7

**Published:** 2014-10-10

**Authors:** Kun Wu, Hongyan Liu, Minmin Yang, Ye Tao, Huihui Ma, Wenxiong Wu, Yang Zuo, Yingzhong Zhao

**Affiliations:** Key Laboratory of Biology and Genetic Improvement of Oil Crops, Ministry of Agriculture, Sesame Genetic Improvement Laboratory, Oil Crops Research Institute of the Chinese Academy of Agricultural Sciences (OCRI-CAAS), Wuhan, Hubei 430062 China; Shanghai Major Biological Medicine Technology Co., Ltd., Shanghai, 201203 China; Fuyang Academy of Agricultural Sciences, Fuyang, Anhui 236065 China

**Keywords:** Genetic map, QTLs, RAD-seq, RIL, Sesame, Grain yield-related traits

## Abstract

**Background:**

Sesame (*Sesamum indicum* L., 2n = 26) is an important oilseed crop with an estimated genome size of 369 Mb. The genetic basis, including the number and locations of quantitative trait loci (QTLs) of sesame grain yield and quality remain poorly understood, due in part to the lack of reliable markers and genetic maps. Here we report on the construction of a hitherto most high-density genetic map of sesame using the restriction-site associated DNA sequencing (RAD-seq) combined with 89 PCR markers, and the identification of grain yield-related QTLs using a recombinant inbred line (RIL) population.

**Result:**

In total, 3,769 single-nucleotide polymorphism (SNP) markers were identified from RAD-seq, and 89 polymorphic PCR markers were identified including 44 expressed sequence tag-simple sequence repeats (EST-SSRs), 10 genomic-SSRs and 35 Insertion-Deletion markers (InDels). The final map included 1,230 markers distributed on 14 linkage groups (LGs) and was 844.46 cM in length with an average of 0.69 cM between adjacent markers. Using this map and RIL population, we detected 13 QTLs on 7 LGs and 17 QTLs on 10 LGs for seven grain yield-related traits by the multiple interval mapping (MIM) and the mixed linear composite interval mapping (MCIM), respectively. Three major QTLs had been identified using MIM with R^2^ > 10.0% or MCIM with h_a_^2^ > 5.0%. Two co-localized QTL groups were identified that partially explained the correlations among five yield-related traits.

**Conclusion:**

Three thousand eight hundred and four pairs of new DNA markers including SNPs and InDels were developed by RAD-seq, and a so far most high-density genetic map was constructed based on these markers in combination with SSR markers. Several grain yield-related QTLs had been identified using this population and genetic map. We report here the first QTL mapping of yield-related traits with a high-density genetic map using a RIL population in sesame. Results of this study solidified the basis for studying important agricultural traits and implementing marker-assisted selection (MAS) toward genetic improvement in sesame.

**Electronic supplementary material:**

The online version of this article (doi:10.1186/s12870-014-0274-7) contains supplementary material, which is available to authorized users.

## Background

Sesame (*Sesamum indicum* L.) is an important and ancient oilseed crop [1]. It is a diploid species (2n = 26) with an estimated genome size of 369 Mb [2]. Sesame seed has the highest oil contents compared with rapeseed, peanut, soybean and other oilcrops [3]. It is also rich in proteins, vitamins and specific antioxidants such as sesamin and sesamolin [4,5], making it one of the best choices for health foods. As the market demand of sesame seeds is rapidly growing, it becomes one of the most important goals to stably improve grain yield of sesame by genetic approaches. Grain yield of sesame per plant is considered to be composed of three components, i.e. the number of capsules per plant, the number of grains per capsule and the grain weight. Some other factors, including plant height, length of capsules (floral) and axis height of the first capsule were found to strongly associated with grain yield of sesame [6]. Since the grain yield-related traits are inherited quantitatively and governed by multiple genes sensitive to the environment, QTL-mapping is needed to dissect the genetics of these traits [7]. The high-density genetic map had been proved to be a very effective and important approach for QTLs detection in rice [8-11] and other crops [12-14]. Unfortunately, there are no yield-related QTLs or genes have been reported in sesame due in part to the lack of reliable DNA markers and genetic maps constructed based on permanent populations.

The first genetic linkage map of sesame was constructed using an F_2_ population derived from the intervariety cross of ‘COI1134’ (white seed coat) and ‘RXBS’ (black seed coat) [15]. This map was 936.72 cM in genetic length with an average marker distance of 4.93 cM. It contained 220 markers, including 8 expressed sequence tag-simple sequence repeats (EST-SSRs), 25 amplified fragment length polymorphism (AFLPs) and 187 Random Selective Amplification of Microsatellite Polymorphic Loci (RSAMPLs), that are distributed on 30 linkage groups, which is more than 2 folds the number of chromosomes of the haploid sesame genome. Later, 14 more genic-SSRs developed from RNA-seq were integrated onto this map [16]. More recently, this map was improved substantially by placement of more markers using an enlarged F_2_ population [17]. This reduced the number of LGs to 14, only one LG more than the haploid chromosome number of sesame. The genetic length of this new map was 1,216 cM, and the marker density was 1.86 cM per marker interval. Four QTLs controlling seed coat color with a heritability ranging from 59.33% to 69.89% were detected in F_3_ populations.

The emergence of massively-parallel, next-generation sequencing (NGS) platforms with continually reducing costs offers unprecedented opportunities for genome-wide marker development and genotyping by sequencing (GBS). Several NGS methods are combined with restriction enzyme digestion to reduce the complexity of the target genomes, making the sequencing load and cost significantly declined [18], while still capable of discovering thousands of single-nucleotide polymorphisms (SNPs) or insertion-deletions (InDels) markers [19-21]. The restriction-site associated DNA sequencing (RAD-seq) was one of the NGS methods that sequencing only the DNA flanking specific restriction enzyme sites to produce a reduced representation of genome, which ligated an adapter containing multiplex identifiers (MIDs) in the reduced-representation libraries (RRLs) [22-27]. In these ways, several high-density genetic maps have been constructed in eggplant [28], ryegrass [13], barley [14], grape [27] and even sesame [29]. Recently, a high-density genetic map of sesame was constructed based on an F_2_ population using the specific length amplified fragment sequencing (SLAF-seq) technology, which is an enhanced RRL sequencing strategy for *de novo* SNP discovery from large populations [21,29]. This map comprises 1,233 SLAF markers that are distributed on 15 linkage groups (LGs), and is 1,474.87 cM in length with average marker spacing of 1.20 cM. Collectively, all the three published sesame genetic maps are not ideal for quantitative traits mapping as they are all on the basis of a temporary population (F_2_) that renders repeated phenotyping unfeasible [30]. Moreover, these maps are not comparable as they lack common markers.

In this study, we identified three thousand seven hundred and sixty-nine pairs of SNP markers through RAD-seq of two sesame varieties ‘Zhongzhi 14’ and ‘Miaoqianzhima’. These markers combined with 1,195 previously reported EST-SSR or genomic-SSR and 79 InDel markers [31], were used to construct a high-density genetic map of sesame using a recombinant inbred line (RIL) population. We further present the identification of grain yield-related QTLs based on these novel genomic resources.

## Results

### RAD sequencing, SNPs and InDels discovery

A total of 62.57 Gb high-quality sequence data containing 312,829,823 pair-end reads was obtained. The read number for the 224 RILs ranged from 598,119 to 3,483,606 with an average of 1,644,718. For the two parents, 3,030,776 reads were from the female parent and 3,881,579 reads were from male parent. After, the number of RAD-tags identified from the male and female parents was 231,000 and 207,000, respectively. The average coverage for individual tag was 16.80-fold in the male parent and 14.64-fold in the female parent. The number of comparable RAD-tags between the two parents was 47,247. However, only 3,769 SNP had been identified for two parents of the RIL population. Most of these SNPs were transition type SNPs with Y(T/C) and R(G/A) types accounting for 30.43% and 30.78%, respectively (Additional file 1). Besides SNPs, 97 InDels (≥2 bp) were identified with 79 successfully designed for further PCR verification and population genotype analysis [31].

Combined with previously published sesame SSRs, a total of 1061 EST-SSRs, 134 genomic-SSRs and 79 InDels were surveyed on the genomic DNA of the two parents. Eighty-nine of these PCR markers detected polymorphism including 44 EST-SSRs, 10 genomic-SSRs and 35 InDels. The efficiencies of EST-SSRs, genomic-SSRs, InDels and SNPs markers in detecting polymorphism between parents varied from 5.0% with EST-SSRs to 46.7% with InDels. All of these polymorphic SSR and InDel markers detected codominant loci.

### Genetic mapping

Before genetic mapping of these markers, 656 SNP markers and 1 InDel marker that had more than 40% missing data in the RIL population were excluded. Another 1,786 SNPs, 15 InDels, 24 EST-SSRs and 4 genomic-SSRs were also excluded for their excessively distorted pattern with segregation ratios of the minor allele frequency less than 0.29. Therefore, a final set of 1,327 SNPs, 19 InDels and 26 SSRs, which mostly inherited in a codominant manner, were used for genetic map construction (Table 1).

**Table 1:** **Summary of markers surveyed for genetic mapping**

**Type**	**Series code**	**No. of markers or tags**	**Number of markers**						**Source**
			**With clear bands**	**Detected polymorphism**	**Excessively missed** ^**a**^	**Excessively distorted** ^**b**^	**Used for mapping** ^**c**^	**Mapped**	
Genomic-SSR	GB, GSSR	134	107	10	0	4	6	6	Dixit et al. [32]; Cho et al. [33]; Spandana et al. [34]
EST-SSR	ZHY, HS, ZM, SEM, Y, SBM	1,061	872	44	0	24	20	16	Wei et al. [15]; Zhang et al. [16]; Yue et al. [35]; Wei et al. [36]; Wang et al. [37]; Yepuri et al. [38]; Wu et al. [31]
InDel	SBI	79	75	35	1	15	19	18	Wu et al. [31]
SNP	SBN	47,247	-	3,769	656	1,786	1,327	1,190	Authors’ laboratory
Total	-	-	-	3,858	657	1,829	1,372	1,230	-

^a^Number of excessively missed markers with more than 40% missing data in population; ^b^Number of excessively distorted markers with segregation ratios of the minor allele frequency less than 0.29; ^c^Number of markers used for genetic mapping without excessively missed or distorted.

**Figure 1:**
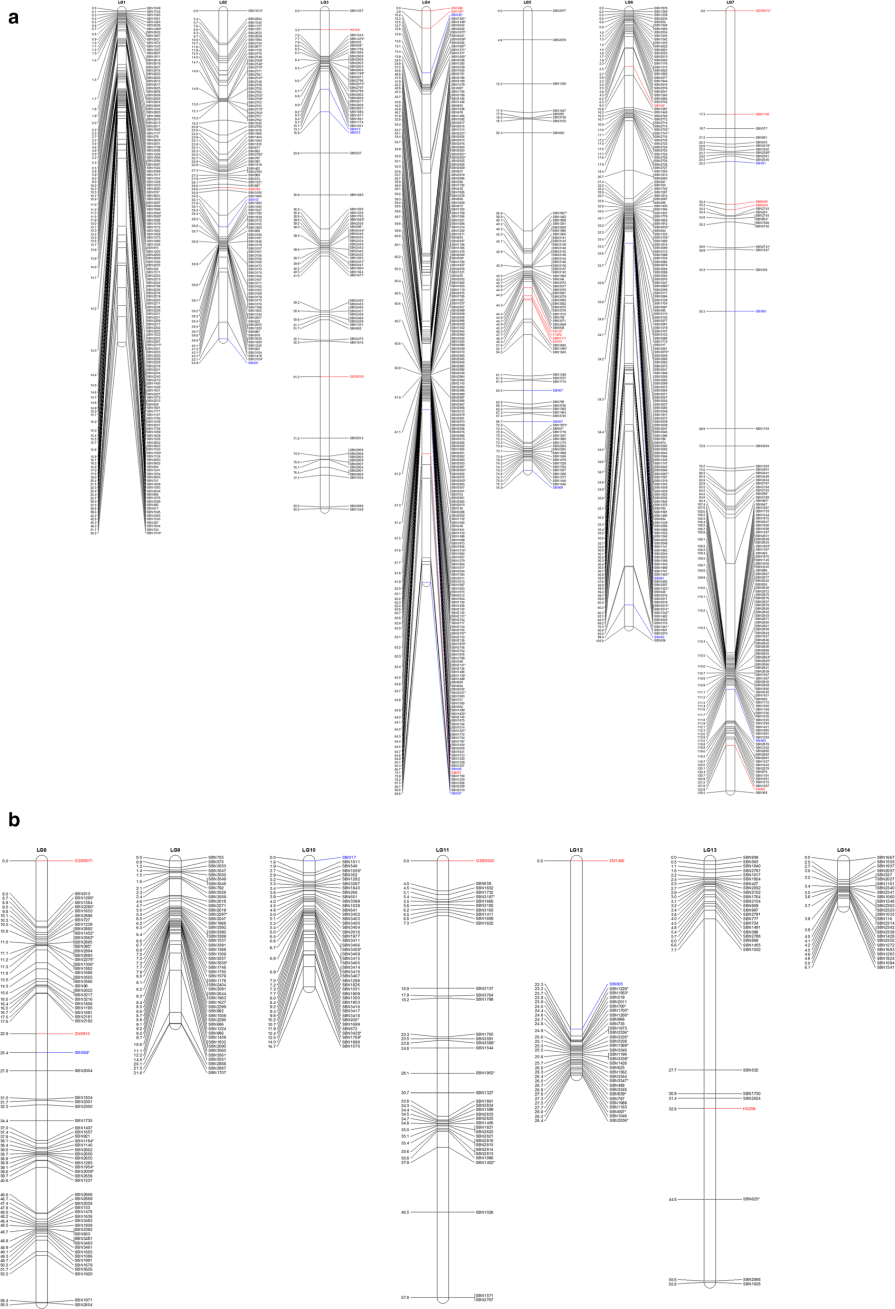
**The high-density genetic map of sesame. a** Linkage groups 1 to 7. **b** Linkage groups 8 to 14. Numbers to the left of each LG are marker positions (cM). The SNP, SSR and InDel markers on the map are in black, red and blue, respectively. The segregation distorted markers on the map are represented by *asterisks* next to the marker locus name.

As a result, 1,230 markers, including 1,190 SNPs, 22 SSRs and 18 InDels were mapped onto 14 different LGs, covering 844.46 cM of the sesame genome and giving an average distance of only 0.69 cM between adjacent markers (Figure 1, Additional file 2). The length of individual LGs varies from 6.08 cM to 130.52 cM, with the average marker distance per LG ranging from 0.23 cM to 1.92 cM and the marker number per LG from 26 to 227 (Table 2). There were 16 gaps more than 10 cM distributed on 9 LGs, excluding LG2, LG8, LG9, LG10 and LG14, with the largest gap of 22.54 cM located on LG6. Most of these gaps were located near the end of the linkage groups (Figure 1), which was considered a reflection of high levels of recombination at distal regions of chromosomes [39,40]. Furthermore, the distributions of SSR, InDel and SNP markers toward different LGs are random, with less than 10% SSR or InDel markers each LGs.

**Table 2:** **Distribution of mapped markers on the 14 linkage groups of sesame**

**Linkage group**	**Number of markers** ^**a**^				**Length (cM)**	**Average distance (cM)**	**Largest gap (cM)**	**No. of gaps >10 cM**	**No. of SDRs** ^**b**^
	**Total**	**SNP**	**SSR**	**InDel**					
LG1	152(7)	152(7)	0	0	55.54	0.37	11.26	1	0
LG2	101(11)	98(11)	1	2	54.9	0.54	8.38	0	1
LG3	77(9)	73(8)	2(1)	2	83.25	1.08	10.3	1	0
LG4	227(24)	220(22)	4(1)	3(1)	95.58	0.42	13.38	3	1
LG5	78(4)	71(4)	4	3	76.88	0.99	16.44	1	0
LG6	183(14)	180(13)	1(1)	2	102.99	0.56	22.54	2	1
LG7	120(10)	112(9)	5(1)	3	130.52	1.09	19.56	3	0
LG8	72(11)	69(10)	2	1(1)	58.45	0.81	7.96	0	0
LG9	50(2)	50(2)	0	0	21.62	0.43	5.6	0	0
LG10	44(5)	43(5)	0	1	16.73	0.38	2.73	0	0
LG11	38(4)	37(4)	1	0	57.79	1.52	11.22	1	0
LG12	33(13)	31(13)	1	1	28.38	0.86	22.26	1	1
LG13	29(1)	28(1)	1	0	55.75	1.92	20.05	3	0
LG14	26(0)	26	0	0	6.08	0.23	2.49	0	0
Total	1230(115)	1190(109)	22(4)	18(2)	844.46	0.69	-	16	4

^a^The number of segregation distortion markers are given in parentheses; ^b^SDR means segregation distortion region.

One thousand one hundred and fifteen mapped markers segregated in the expected 1:1 ratio in the population. However, segregation of 115 mapped markers, including 4 SSRs, 2 InDels and 109 SNPs, were significantly deviated from this ratio (*P* <0.05) (Table 2). Seventy-seven (61.1%) segregation distorted markers exhibited skewed genotypic frequencies toward ‘Zhongzhi 14’, while 49 (38.9%) toward ‘Miaoqianzhima’. Most of these markers have no effect on the calculation of map distance, except SBN1614, SBN3567 and GSSR074. Compared to mapped SNP markers and InDel markers, the mapped SSR markers had the highest percentage of skewed markers at 17.4%. These segregation distortion markers were distributed on 13 LGs, excepting LG14. The largest LG4 with 227 mapped markers had the most segregation distortion markers. The frequency of segregation distortion marker on LG12 was much higher than for other LGs at 39.4%. Four regions of segregation distortion (SDR) were detected on four LGs, including LG2, LG4, LG6 and LG12 (Table 2). Most of these SDRs distributed near the end of their LGs, with 3 to 5 skewed markers each and accounting for 14.3% of the total skewed markers in the map. Most skewed markers in four SDRs were SNP type, with one EST-SSR marker (ZM1197) and one InDel marker (SBI035) in SDR-LG4. All the markers in SDR-LG2, SDR-LG6, and SDR-LG12 exhibited skewed genotypic frequencies towards ‘Zhongzhi 14’, while towards ‘Miaoqianzhima’ in SDR-LG4.

### Phenotypic analysis

**Figure 2:**
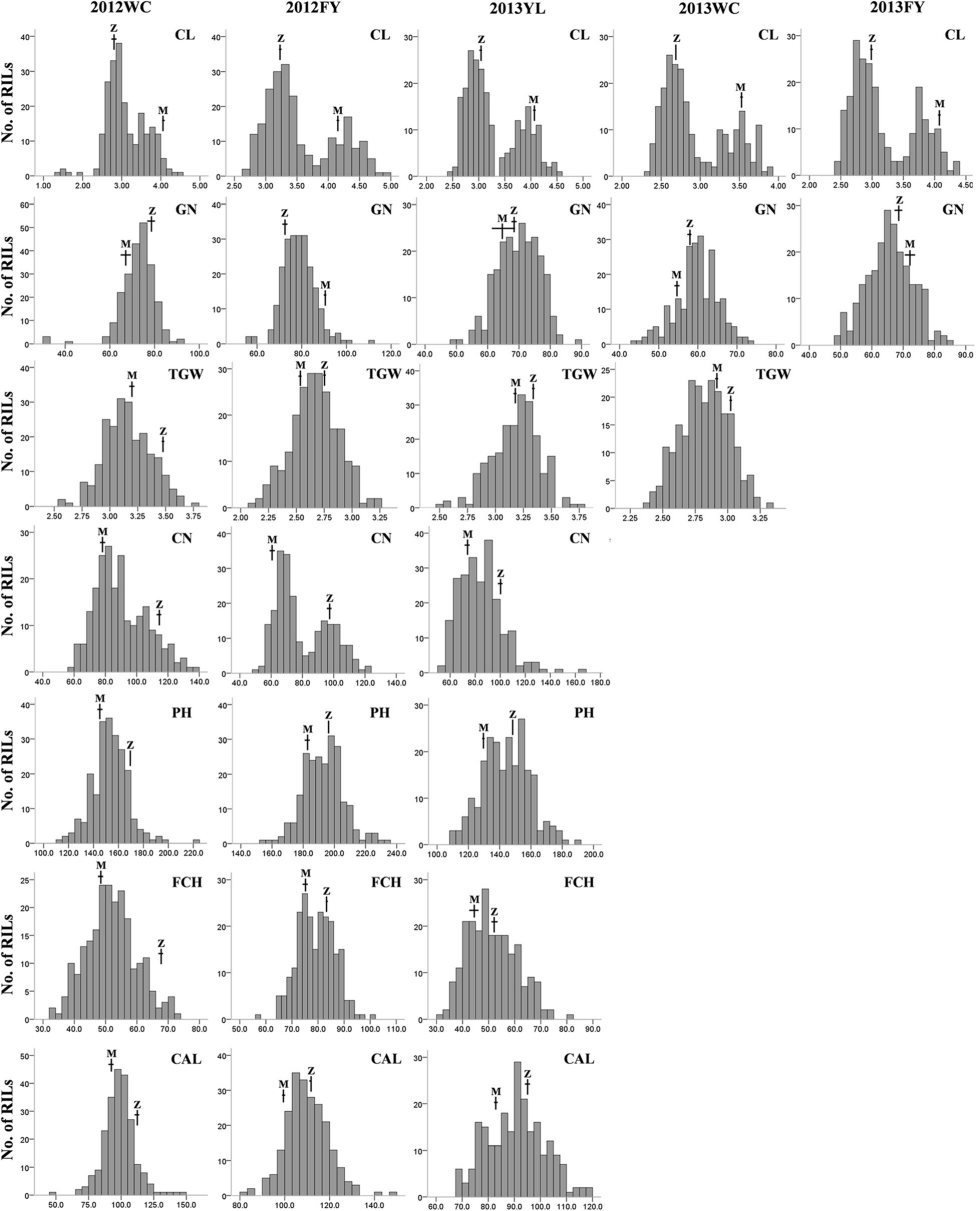
**Distributions of the phenotypic data in the ‘Miaoqianzhima × Zhongzhi 14’ RIL population.** PH, plant height; FCH, first capsule height; CAL, capsule axis length; CN, capsule number per plant; CL, capsule length, GN, grain number per capsule; TGW, thousand grain weight. Mean and standard deviation of two parents are indicated at the top of each histogram, with *Z* and *M* representing Zhongzhi 14 and Miaoqianzhima, respectively.

In all experiments, seven yield-related traits showed significant differences between the mapping parental lines. Compared to Miaoqianzhima, the male parent Zhongzhi 14 displayed significantly taller plant height (PH), shorter first capsule height (FCH), longer capsule axis length (CAL), more capsule number per plant (CN), shorter capsule length (CL) and larger thousand grain weight (TGW) (Figure 2). The PH, FCH, CAL and TGW in 2013FY or 2013WC were missed for their bad field performance caused by extreme weathers. Interestingly, the average grain number per capsule (GN) of Zhongzhi 14 was more than Miaoqianzhima in Wuchang (2012WC, 2013WC), while less in Fuyang (2012FY and 2013FY). All traits showed a continuous distribution and transgressive segregation in the RIL population (Figure 2), indicating governed by multiple genes. The near-normal curve distribution of PH, FCH, CAL, GN and TGW suggested a polygene mode of the genetic control; but CL and CN showed a bimodal distribution, suggesting the involvement of major effect genes. Analysis of variance (ANOVA) showed that the between-line variations of all traits in each trial were significant at *P* = 0.001. The broad-sense heritability of the seven traits ranged from 29.8% (FCH) to as high as 95.7% (CN) (Table 3). The heritabilities of each trait are in line with their corresponding distributions.

**Table 3:** **QTLs for grain yield-related traits and their epistasis detected by MCIM from the analysis of the RILs in multi-trials**

**Traits**	**QTL**	**LG**	**Marker interval**	**QTL region (cM)**	**QTL peak position**	**Additive effect** ^**a**^	**h**_***a***_^**2**^ **(%)** ^**b**^	***ae*** ^**a**^	**h**_***ae***_^**2**^ **(%)** ^**b**^	***H*** ^***2***^ **(%)** ^**c**^
Plant height	*Qph-6*	LG6	SBN3089-SBN3112	33.5-33.8	33.5	3.0724***	3.63			32.5
	*Qph-12*	LG12	ZM1466-SBI005	13.5-22.3	22.0	2.8852***	3.36			
First capsule height	*Qfch-4*	LG4	SBN3000-SBN1825	60.7-60.8	60.8	2.0016***	4.72			29.8
	*Qfch-11*	LG11	SBN1622-SBN3137	8.3-17.9	13.3	2.1111***	5.02			
	*Qfch-12*	LG12	ZM1466-SBI005	12.0-22.3	19.0	2.0667***	3.37			
Capsule axis length	*Qcal-5*	LG5	SBN3577-SBN3576	43.7-44.4	43.9	1.7741***	2.54			69.7
	*Qcal-9*	LG9	SBN3559-SBN2018	2.1-4.6	3.4	1.7761***	1.99			
Capsule number per plant	*Qcn-11*	LG11	SBN1622-SBN3137	11.3-17.9	15.3	−4.1764***	4.48			95.7
Thousand grain weight	*Qtgw-11*	LG11	SBN1798-SBN1765	18.2-20.2	19.2	0.0638***	5.78			48.9
Grain number per capsule	*Qgn-1*	LG1	SBN1076-SBN2389	29.7-36.0	34.7	1.2248***	1.82			54.6
	*Qgn-6*	LG6	SBN1261-SBN1801	88.3-92.9	92.3	1.7740***	5.61	−0.8819*	1.16	
	*Qgn-12*	LG12	SBN1362-SBN3344	26.0-26.7	26.3	−1.4724***	4.26			
Capsule length	*Qcl-3*	LG3	SBN2902-SBN1034	76.1-77.4	76.4	−0.0857***	3.13			86.8
	*Qcl-4*	LG4	SBN2166-SBN1014	64.1-64.2	64.1	0.0653***	3.02			
	*Qcl-7*	LG7	SBN3401-SBN3441	73.8-79.0	77.0	0.0529***	1.93			
	*Qcl-8*	LG8	SBN1686-SBN3565	11.0-11.2	11.1	0.0420***	1.70			
	*Qcl-12*	LG12	ZM1466-SBI005	14.0-18.0	16.0	−0.4237***	45.39			
Trait	Epistatic interaction		Nearest marker	QTL peak position (cM)		*aa* ^**a**^	h_*aa*_^2^(%)^**b**^			
First capsule height	*Qfch-4* and *Qfch-12*		SBN3000 and SBI005	60.8 and 19.0		1.2998***	1.59			

^a^Positive and negative values indicated additive effect, additive × environment interaction effect (*ae*) or epistatic interaction additive effect (*aa*) by the alleles of Zhongzhi 14 and Miaoqianzhima, respectively; ^b^Contibution ratio of QTL additive effect, additive × environment interaction effect (*ae*) or epistatic interaction additive effect (*aa*); *, **, *** Significant at 0.05, 0.01, 0.001 probability levels, respectively; ^c^The broad-sense heritability (*H* ^*2*^) was calculated with the formula *H* ^2^ = *σ*_*g*_^2^/(*σ*_*g*_^2^ + *σ*_*e*_^2^/*r*).

Trial-wide correlation coefficients of all seven traits were significant at the level of *P* =0.01 (Additional file 3). Correlation of CL among different environments (years or locations) were strong with the coefficients above 0.80, while much weaker correlation for CAL were noted with the coefficients ranging from 0.27 to 0.35. Across the three environments where phenotypic data were available (2012WC, 2012FY and 2013YL), significant positive correlations were observed between PH and FCH (*P* ≤0.01), PH and CAL (*P* ≤0.01), PH and TGW (*P* ≤0.05), FCH and TGW (*P* ≤0.05), even CL and GN (*P* ≤0.01), while significant negative correlation were observed between CN and TGW (*P* ≤0.05) (Table 4). More interestingly, GN and TGW were positively correlated in 2012FY (*P* ≤0.01), but negatively correlated in 2013YL (*P* ≤0.01).

**Table 4:** **The pairwise correlation coefficients between different traits in three environments**

	**Trait**	**PH**	**FCH**	**CAL**	**CN**	**CL**	**GN**	**TGW**
2012WC	PH	1						
	FCH	0.587**	1					
	CAL	0.574**	−0.063	1				
	CN	0.401**	−0.075	0.435**	1			
	CL	0.236**	0.131*	0.154*	0.039	1		
	GN	0.412**	0.320**	0.148*	0.108	0.485**	1	
	TGW	0.141*	0.147*	0.161**	−0.113*	0.175**	−0.095	1
2012FY	PH	1						
	FCH	0.684**	1					
	CAL	0.848**	0.224**	1				
	CN	−0.214**	−0.455**	−0.01	1			
	CL	−0.104	−0.025	−0.101	−0.271**	1		
	GN	0.017	0.024	0.017	−0.340**	0.303**	1	
	TGW	0.354**	0.307**	0.311**	−0.524**	0.058	0.217**	1
2013YL	PH	1						
	FCH	0.708**	1					
	CAL	0.749**	0.095	1				
	CN	0.116*	−0.288**	0.407**	1			
	CL	−0.044	−0.122*	0.042	−0.244**	1		
	GN	0.205**	0.130*	0.189**	−0.197**	0.401**	1	
	TGW	0.264**	0.277**	0.109	−0.256**	−0.046	−0.160**	1

*Significant at *P* ≤0.05, **Significant at *P* ≤0.01.

### QTL analysis

A total of 13 yield-related QTLs were found on 7 linkage groups using the multiple interval mapping (MIM) methods. A range of one to three QTLs were detected for individual traits (Table 5). Six QTLs were detectable in more than one trial, including *Qph-12*, *Qtgw-11*, *Qgn-1*, *Qgn-6*, *Qgn-12* and *Qcl-12*, while others were repeatable by two softwares. Most of them showed positive additive effects by the alleles of Zhongzhi 14 except *Qgn-12* and *Qcl-12.* Six major-effect QTLs were detected with the phenotypic effect (R^2^) more than 10%, including one QTL, *Qcl-12*, showing R^2^ ranged from 52.2% to 75.6%.

**Table 5:** **QTLs of yield-related traits detected by MIM from the analysis of the RILs in five trials**

**Traits**	**Trials**	**QTL**	**LG**	**LOD threshold** ^**a**^	**Marker Interval**	**QTL region (cM)**	**QTL peak position**	**LOD**	**R** ^**2**^ **(%)** ^**b**^	**Additive effect** ^**c**^
Plant height	2013YL	*Qph-6*	LG6	3.1	SBN1813-SBN3112	26.7-33.1	32.5	3.31	6.0	4.0183
	2012WC	*Qph-12*	LG12	3.0	ZM1466-SBN1229	21.9-23.0	22.3	3.10	5.6	3.5444
	2013YL	*Qph-12*	LG12	3.2	ZM1466-SBN1229	12.1-23.3	19.0	3.92	9.1	4.9657
First capsule height	2013YL	*Qfch-4*	LG4	3.2	SBN693-SBI050	60.0-66.7	60.1	3.50	6.2	2.3771
	2012WC	*Qfch-11*	LG11	3.1	SBN1609-SBN3137	6.7-16.9	13.3	4.20	8.2	2.5115
	2013YL	*Qfch-12*	LG12	3.2	ZM1466-SBI005	6.0-23.7	19.0	5.39	11.5	3.2616
Capsule axis length	2012FY	*Qcal-5*	LG5	3.0	SBN1595-SBM1111	43.0-48.0	43.9	4.40	8.1	4.2033
	2013YL	*Qcal-9*	LG9	3.1	SBN3559-SBN2018	2.4-4.7	3.4	3.86	9.2	3.5580
Capsule number per plant	2013YL	*Qcn-11*	LG11	3.0	SBN1622-SBN3137	14.3-16.9	16.3	3.29	7.0	−4.7757
Thousand grain weight	2012WC	*Qtgw-11*	LG11	3.2	SBN1798-SBN1765	17.9-19.2	18.2	4.13	7.7	0.0618
	2013YL	*Qtgw-11*	LG11	3.2	SBN1798-SBN1765	18.2-20.2	19.2	3.68	9.2	0.0672
	2013WC	*Qtgw-11*	LG11	3.0	SBN1798-SBN1765	17.9-21.2	19.2	5.14	12.3	0.0695
Grain number per capsule	2013WC	*Qgn-1*	LG1	3.1	SBN2389-SBN297	36.8-48.2	46.1	3.90	6.8	1.4556
	2013FY	*Qgn-1*	LG1	3.2	SBN1076-SBN1844	30.4-46.4	39.6	6.30	11.0	2.4169
	2012WC	*Qgn-6*	LG6	3.0	SBN1261-SBI043	78.9-99.0	92.3	4.40	8.0	2.2658
	2012FY	*Qgn-6*	LG6	3.1	SBN1261-SBI043	83.2-99.0	92.9	6.9	11.4	2.3877
	2013YL	*Qgn-6*	LG6	3.1	SBN1261-SBI043	74.4-99.0	89.5	8.3	18.3	2.9494
	2013WC	*Qgn-12*	LG12	3.1	SBI005-SBN3344	22.3-26.7	26.0	5.0	7.9	−1.5765
	2013FY	*Qgn-12*	LG12	3.2	SBI005-SBN3344	22.3-26.7	25.3	8.3	13.6	−2.7619
Capsule length	2012WC	*Qcl-12*	LG12	5.0	ZM1466-SBI005	3.0-22.3	18.0	29.55	52.2	−0.3805
	2012FY	*Qcl-12*	LG12	5.0	ZM1466-SBI005	3.0-22.3	17.0	42.80	70.3	−0.5104
	2013YL	*Qcl-12*	LG12	5.0	ZM1466-SBI005	3.0-22.3	17.0	50.56	72.0	−0.4851
	2013WC	*Qcl-12*	LG12	5.0	ZM1466-SBI005	3.0-22.3	17.0	54.92	74.0	−0.3964
	2013FY	*Qcl-12*	LG12	5.0	ZM1466-SBI005	3.0-22.0	17.0	56.20	75.6	−0.4955

^a^LOD thresholds determined by 1,000 permutation; ^b^Proportion of phenotypic variation explained by individual QTL; ^c^Positive and negative values indicated additive effect by the alleles of Zhongzhi 14 and Miaoqianzhima, respectively.

QTL mapping was also performed with QTLNetwork 2.0 under the mixed linear composite interval mapping (MCIM) algorithm to dissect the main additive effects (*a*), the additive-additive epistatic effects (*aa*) and the additive-environmental interaction effects (*ae*) in multi-trials. A total of 17 QTLs were detected on 10 linkage groups (Table 3). All of them had significant *a* effects, and *Qgn-6* also had significant *ae* effects at *P* ≤0.05 in 2013FY. All of them showed significant additive effect at *P* ≤0.001, and explained 1.70-45.39% of the phenotype variation with four major QTLs larger than 5.0%. Two QTLs for first capsule height, *Qfch-4* and *Qfch-12*, were also detected with significant *aa* effect explained 1.59% of the phenotypic variation (Table 3).

We also compared QTLs that both identified using MIM and MCIM for seven different yield-related traits. Thirteen QTLs were detected by two methods with similar QTL regions, while *Qcl-3*, *Qcl-4*, *Qcl-7* and *Qcl-8* were only detected by MCIM. Three major-effect QTLs were detected by two methods with R^2^ > 10.0% or h_a_^2^ > 5.0%, including *Qtgw-11*, *Qgn-6* and *Qcl-12*. Furthermore, the *Qph-12* and *Qfch-12*, contributed by Zhongzhi 14, and *Qcl-12* contributed by Miaoqianzhima, were co-located. Three QTLs, *Qfch-11* and *Qtgw-11* contributed by Zhongzhi 14, and *Qcn-11* contributed by Miaoqianzhima, were located closely on linkage group LG11.

## Discussion

### Construction of a high-density genetic map in sesame

In this study, only 44 (5.0%) EST-SSRs and 10 (9.3%) genomic-SSRs were found polymorphic in the mapping population and thus were useful for genetic map construction. This rate of polymorphism is much lower than in many previous reports in sesame [16,32,34], indicating a narrower genetic dissimilarity between the parents. However, thanks to the high-throughput RAD-Seq technology, we were able to discover more than 3000 SNPs plus dozens of InDels from ~40 k comparable RAD-tags. The rate of SNPs was 7.98% across the genome, which was higher than 5.12% reported by Zhang et al. [29]. The observation that most SNPs belong to the Y(T/C) (30.43%) and R(G/A) (30.78%) types are consistent with the situations previously reported in sesame [29] and other species including even human [41].

Furthermore, the mapping population in this study was the first reported and the largest permanent mapping population in sesame. Compared to other published genetic maps in sesame, the map constructed in this paper had the highest marker density, the similar number of linkage groups compare to *Sesamum indicum* L. chromosomes (2n = 26), fewer distortion markers, fewer and smaller gaps [15,17,29]. Furthermore, 2,442 (64.8%) SNP markers and 44 (49.4%) polymorphic PCR markers that excessively missed or distorted were excluded for map construction in this study, while more than 65.4% markers were discarded for their unexpected segregation patterns that reported by Zhang et al. [29]. There were also 115 (9.35%) markers that showed significant segregation distortion (*P* <0.05) were mapped onto our map, while 205 (16.63%) [29] and 79 (10.91%) [17] on other two genetic maps in sesame. Four SDRs were detected on 4 LGs of our map, while 18 SDRs on 11 LGs of SLAF map [29]. Most of them distributed near the end of LGs, and may be involved in gametic, zygotic or other selections [42,43]. The map size reported here is 844.46 cM, which is significantly shorter than previously published maps of 1,216 and 1,474 cM. This might be due to the discarded linkage groups with less than 20 markers and the fewer segregation distortion markers and SDRs in our map. More importantly, several PCR markers on our map will be very useful information for the comparison of maps, genes or QTLs reported in sesame. Therefore, the high-density genetic map constructed in this study combined the advantages of two older maps in sesame, and will be an ideal map for QTL/gene mapping, comparative genomics analysis, map-based cloning and so on. However, it should be pointed out that the utility as a general tool for the research community has limitations for the genetic map presented is mainly based on SNP between only two sesame varieties and the SNP flanking sequence is only 85 bp.

### Identification of grain yield-related QTLs using high-density genetic map in sesame

As grain yield is a complex quantitative trait controlled by multiple genes and sensitive to environments, it is imperative to phenotype yield-related traits repeatedly for reliable QTL mapping. Here the availability of a permanent segregating population (the RIL) makes it feasible for repeated phenotyping both over time and location. Since significantly (*P* = 0.01) correlations were found for each trait among different environments, the field experiments must have provided reliable phenotypic data for QTL mapping. However, trial-wide correlation coefficients below 0.351 for CAL or below 0.509 for CN indicated a weak or moderate correlation, respectively. And three QTLs for CAL and CN were identified in only one environment, although be detected using both MIM and MCIM.

Finally, thirteen yield-related QTLs on 7 LGs and 17 QTLs on 10 LGs had been detected using MIM and MCIM method, respectively. These were the first reported grain yield-related QTLs in sesame, and all of them were detectable in more than one trial or by two algorithms. The genetic control of seven yield-related traits was mostly comprised of few major QTLs plus several minor QTLs. Three major QTLs had been detected using MIM with R^2^ > 10.0% or MCIM with h_a_^2^ > 5.0%. Ten minor QTLs had been identified for seven yield-related traits using both MIM and MCIM. On the other hand, we found a QTL (*Qgn-6*) showed significant *ae* effect, and one pair of QTLs for FCH with significant *aa* effect. Several *ae* or *aa* effect of yield-related QTLs also had been reported in wheat [44], soybean [45], oilseed rape [46], and so on. These QTLs with *a*, *ae* or *aa* effect will be very important common and special information for yield improvement in sesame.

Furthermore, significantly correlations were found among some of the yield-related traits, which are indicative of closely linked or pleiotropic genetic factors controlling these traits. This was then verified by co-localization of several QTLs for these traits. The co-localization of *Qph-12* and *Qfch-12*, all from the Zhongzhi 14 alleles, were in line with the significant positive correlation between PH and FCH. The positive correlation was found between FCH and TGW, but negative correlation between CN and TGW or CN and FCH. Correspondingly, *Qfch-11* and *Qtgw-11* with positive additive effect from Zhongzhi 14 alleles, and *Qcn-11* with negative additive effect from Miaoqianzhima alleles, were closely located on LG11. Nevertheless, not all correlations can be explained by QTL co-localization, such as CL and GN, PH and CN. These contradictions could be due to the effect of undetected QTLs or reasons other than pleiotropy or linkage.

### Future perspectives and challenges in sesame breeding

Improvement of yield is one of the most important targets for sesame breeding; however, it is a time-consuming and tedious project because multiple complex and environment-sensitive components are involved in this process. The identification of yield-related QTLs in this study has laid a preliminary foundation for marker assisted selection (MAS) toward the yield traits in sesame. Even though, for some minor QTLs with low LOD scores, further validation is necessary before utilizing them in breeding. On the other hand, the epistatic interaction and the co-location of yield-related QTLs may be beneficial or problematic for pyramiding of desired loci, depending on their patterns. The positive *aa* effects of *Qfch-4* and *Qfch-12* indicate that the integration of both QTLs will be beneficial to the improvement of FCH in this study. The closely located *Qtgw-11* and *Qcn-11* showed significant additive effect on TGW and CN, but the favorable alleles are carried by different parent lines. Thus, there are still a lot of efforts to make to precisely dissect the linked or epistatic QTLs, or screen for germplasm with independent favorable allelic variations, to facilitate breeding.

In this study, we found that most QTLs showing positive additive effects are from the alleles of Zhongzhi 14, an excellent commercial cultivar with several high-yield characters. However, two identified QTLs for GN and CN contributed by Miaoqianzhima. It means that introduction of these two QTLs using the alleles of Miaoqianzhima will further improve the GN and CN of Zhongzhi 14. Furthermore, we have found ‘the superior line’ predicted using QTLNetwork 2.0 with significantly increased genotype effect for GN value than two parents [47] (data not showed). So there will be very great breeding potential for the improvement of grain number per capsule with this RIL population. This genotyped RIL population combined with high-density genetic map will also serve as an effective study system for characterizing serious of important agricultural traits, such as yield, oil or protein content in grain, stress tolerance, and so on.

## Conclusions

This report presents by far the first QTL mapping work of yield-related traits in sesame using a RIL population, in addition to the construction of a high density genetic map. We developed 3,769 SNPs markers by RAD tag sequencing, and constructed a so far most high-density genetic map of 14 LGs in combination with SSR and InDel markers. Using this RIL population and genetic map, several grain yield-related QTLs had been detected in more than one trials or by both MIM and MCIM method, including three major effect QTLs with R^2^ > 10.0% or h_a_^2^ > 5.0%. Three QTLs with significant *ae* or *aa* effect had also been identified using MCIM algorithm. Several co-localized QTLs were identified that partially explained the correlations among seven yield-related traits. The high-density genetic map and yield-related QTLs in the current study solidified the basis for studying important agricultural traits, map-based cloning of grain yield-related genes and implementing MAS toward genetic improvement in sesame.

## Methods

### Plant materials and field trials

The mapping population used in this study consists of 224 F_8:9_ recombinant inbred lines derived from single-seed descent from a cross between ‘Miaoqianzhima’ and ‘Zhongzhi 14’, both are white seed-coated. The male parent ‘Zhongzhi 14’ is a commercial cultivar grown widely in China while the female parent ‘Miaoqianzhima’ is a landrace accession originating from Anhui province in China. The two varieties are distinct in many morphological traits, including plant height, growth habit, capsule shape, leaf shape and color, as well as resistances to multiple diseases.

Five field trials were set in five environments during the year 2012 to 2013 at normal planting season (from June to September), two in Wuchang (2012WC, 2013WC), two in Fuyang (2012FY, 2013FY), and one in Yangluo (2013YL). Wuchang (30°52’N, 114°32’E) and Yangluo (30°73’N, 114°62’E), which are ~38.6 km apart, both are located in the summer-sown sesame zone of the middle Yangtze Valley, while Fuyang (32°93’N, 115°81’E) in the summer-sown sesame zone of the Huang Huai basin. The aforementioned two zones take up more than 50% of China’s sesame-grown area. All trials were in a randomized complete blocks design, with three replicates each environment. Each plot had two 2.0-m rows spaced 0.4 m apart. At the two-euphylla stage, the plants were thinned and only thirteen evenly distributed plants in each row were retained for further analyses.

### Traits evaluation

In each plot or genotype, only six uniform plants were used for trait evaluation. Plants at the two ends of each row were not selected to avoid edge effects. Traits evaluated include plant height (PH, cm), first capsule height (FCH, cm), capsule axis length (CAL, cm), capsule number per plant (CN), capsule length (CL, mm), grain number per capsule (GN) and thousand grain weight (TGW, g). CAL was measured as the length of axis from the lowest capsule to the top one. CL and GN were measured as the mean values of 18 uniform capsules from six plants. The half of TGW was measured as the mean weight of three independent samples of 500 grains. Other traits were measured as the mean values of 6 plants. All of them were measured just before the harvest stage.

### Genomic DNA extraction and PCR

Genomic DNA was extracted from young leaves using the DNA extraction kit (TIANGEN Co. Ltd, Beijing). One thousand two hundred and seventy-four PCR markers, including 134 genomic-SSRs, 1,061 EST-SSRs and 79 InDels were used for genetic map construction (Table 1) [31]. Polymerase chain reactions (PCR) for SSRs and InDels were performed in 10 μl reactions, containing 10 ng DNA, 2 pmol of each primers, 2 nmol dNTPs, 15 nmol MgCl_2_, 0.2 U Taq DNA polymerase (Thermo Fisher Scientific, America) and 1 × PCR buffer supplied together with the enzyme. The PCR cycles were 94°C 3 min, 36 cycles of 94°C 20 s, 55°C ~ 60°C (depending on the primers) 30 s, 72°C 40 s, and a 5 min at 72°C for final extension. PCR products were separated in 8% non-denaturing polyacrylamide gels (Acr:Bis =19:1 or 29:1) on a constant voltage of 180 V for 2 ~ 3 h, and were visualized by silver staining [48].

### RAD sequencing, InDel and SNP markers development

Restriction-site Associated DNA (RAD) approach combined with Illumina DNA sequencing was used for rapid and effective discovery of InDel and SNP markers. RAD library construction, sample indexing and pooling followed Baird et al. [49]. The restriction enzyme *Eco*R I was used to cut the DNA of two parents and RIL population [50]. 22 multiplexed sequencing libraries were constructed, in which each DNA sample was assigned a unique nucleotide MID for barcoding. Single-end (101 bp) sequencing was performed using Illumina NGS platform HiSeq2000 in a total throughput of 22 lanes.

Raw sequence reads without MID barcode sequences were trimmed to 85 nucleotides from the 3’ end to ensure more than 90% of the nucleotides have a quality value above Q30 (equals 0.1% sequencing error) and more than 99% above Q20 (equals 1% sequencing error). Reads of low quality, including reads with <85 bp after trimming or with ambiguous barcodes, were discarded. For InDels and SNPs calling, the trimmed reads were clustered into RAD-tags based on sequence similarity using *Stacks* under default parameters [51]. Clustered RAD-tags with very high read depth (>500) were excluded [51]. Sequences of RAD-tags were blasted between the two parental plants. InDels (≥2 bp) or SNPs were identified in alignment results, and regarded as true polymorphisms when each allele was observed at least three times. InDel markers were developed for PCR analysis by gaps in alignment results with another protocol [31]. The resultant sequence reads containing SNPs were compared among RIL plants. Only SNPs that were consistently discovered in parents and the progenies were retained [50]. The genotypes of SNP or PCR markers of 224 RILs were used for genetic map construction.

#### Linkage mapping

The marker segregation ratios were examined using the chi-square test. The poorly performing markers were removed before map construction, which excessively missed with more than 40% missing data in the RIL population or excessively distorted with segregation ratios more than of the minor allele frequency less than 0.29 [13]. A region with at least three adjacent loci showing significant segregation distortion (*P* <0.05) was defined as a segregation distorted region (SDR) [52]. The genetic linkage map was constructed using JoinMap 4 (Kyazma, Wageningen, Netherlands). Linkage groups were determined using a minimum LOD value of 5.0 and a maximum recombination of 45%. The regression mapping algorithm was used under the LOD threshold of 3.0 to determine the orders of markers in each linkage group. The linkage groups harboring less than 20 markers were discarded. A ripple was performed after addition of each locus, with the goodness-of-fit jump threshold for removal loci =5.0 and third round = Yes. The Kosambi mapping function was used to translate recombination frequencies into map distances. The final marker order of each linkage group was verified by the software program RECORD [53]. The linkage map was graphically visualized with MapChart 2.2 [54].

#### QTL analysis

The mean phenotypic data of three replicates (blocks) in different trials (environments) from all 224 lines (genotypes) were analyzed for frequency distributions, standard errors, pearsons correlation coefficients and ANOVA using SAS Statistics package [55]. The broad-sense heritability (*H*^*2*^) was calculated with the formula *H*^2^ = *σ*_*g*_^2^/(*σ*_*g*_^2^ + *σ*_*e*_^2^/*r*), where σ_*g*_^*2*^ represents the genetic variance, σ_*e*_^*2*^ is the residual variance, and *r* is the number of replicates per genotype.

QTLs were detected for each of the seven traits using the MIM method implemented in Windows QTL Cartographer 2.5 [56] and MCIM in QTLNetwork 2.0 [57]. In Windows QTL Cartographer 2.5, a Composite interval mapping (CIM) analysis was run at first using Model 6 for one trait in one trial independently, with the forward and backward stepwise regression under a step size of 1 cM and a window size of 10 cM. The LOD significance thresholds (*P* <0.05) were determined by running 1,000 permutations tests [14]. The MIM was subsequently used to more precisely locate the QTLs. The QTL peaks identified in CIM were used as the initial model for the MIM and progressively refined the model using Bayesian Information Criteria (BIC-M0). QTL effects including their percentage of phenotypic variance (total *R*^2^) were estimated with the final model fitted in MIM, and the *R*^2^ for individual QTL was estimated using CIM. The boundaries of the confidence interval of the QTLs were estimated with the positions where the LOD value drop-off was equal to 1 [58].

QTLNetwork 2.0 was also used to identify QTL epistasis and QTL-environment (QE) interactions of one trait in several trials with three replicates together, which employed the genome scan parameters of a 10 cM testing window, 1 cM walk speed and 10 cM filtration window. Two-dimensional (2D) genome scans were carried out to search for multiple interacting QTLs. A genome-wide threshold value of the *F*-statistic (α = 0.01) for declaring the presence of a QTL was estimated by 1,000 random permutations. A Monte Carlo Markov Chain method with Gibbs sample size of 20,000 was used to estimate QTL effects [59]. The sum of individual phenotypic variance explained by each QTL was calculated as the total phenotypic variance explained by all QTL for each trait.

### Availability of supporting data

The raw sequence data of the RAD sequencing have been deposited in the National Center for Biotechnology Information (NCBI) Sequence Read Archive (SRA) database under the accession number SRA100255.

## Additional files

Additional file 1: Table S1. Sequences of SNP markers developed by RAD sequencing in the current study. Additional file 2: Table S2. Map position and population genotype of 1,230 mapped markers. Additional file 3: Table S3. The correlation coefficients of individual traits among different trials.

## Abbreviations

*A*: Additive effects
*aa*: Additive-additive epistatic effects
*ae*: Additive-environmental interaction effects
ANOVA: Analysis of variance
CAL: Capsule axis length
CL: Capsule length
CN: Capsule number per plant
EST: Expressed sequence tag
FCH: First capsule height
GN: Grain number per capsule
InDel: Insertion-deletion
LG: Linkage groups
MCIM: Mixed linear composite interval mapping
MIM: Multiple interval mapping
NGS: Next-generation sequencing
PH: Plant height
QTL: Quantitative trait locus
RAD-seq: Restriction-site associated DNA sequencing
RIL: Recombination inbred line
SDR: Segregation distortion regions
SNP: Single-nucleotide polymorphism
SSR: Simple sequence repeat
TGW: Thousand grain weight

## References

[1] Bedigian D (2003). Evolution of sesame revisited: domestication, diversity and prospects. Genet Resour Crop Ev.

[2] Zhang H, Miao H, Wang L, Qu L, Liu H, Wang Q, Yue M: **Genome sequencing of the important oilseed crop*****Sesamum indicum*****L.** *Genome Biol* 2013, **14**(1):401. 10.1186/gb-2013-14-1-401 PMC3663098 23369264

[3] Anilakumar KR, Pal A, Khanum F, Bawa AS (2010). Nutritional, medicinal and industrial uses of sesame (*Sesamum indicum* L.) seeds-an overview. Agriculturae Conspectus Scientificus (ACS).

[4] Namiki M (1995). The Chemistry and Physiological Functions of Sesame. Food Rev Int.

[5] Moazzami AA, Kamal-Eldin A (2006). Sesame seed is a rich source of dietary lignans. J Am Oil Chem Soc.

[6] Biabani AR, Pakniyat H (2008). Evaluation of seed yield-related characters in sesame (*Sesamum indicum* L.) using factor and path analysis. Pak J Biol Sci.

[7] Morrell PL, Buckler ES, Ross-Ibarra J (2012). Crop genomics: advances and applications. Nat Rev Genet.

[8] Li Y, Fan C, Xing Y, Jiang Y, Luo L, Sun L, Shao D, Xu C, Li X, Xiao J, He Y, Zhang Q (2011). Natural variation in GS5 plays an important role in regulating grain size and yield in rice. Nat Genet.

[9] Yu H, Xie W, Wang J, Xing Y, Xu C, Li X, Xiao J, Zhang Q: **Gains in QTL detection using an ultra-high density SNP map based on population sequencing relative to traditional RFLP/SSR markers.** *PLoS One* 2011, **6**(3):e17595. 10.1371/journal.pone.0017595 PMC3048400 21390234

[10] Marathi B, Guleria S, Mohapatra T, Parsad R, Mariappan N, Kurungara VK, Atwal SS, Prabhu KV, Singh NK, Singh AK: **QTL analysis of novel genomic regions associated with yield and yield related traits in new plant type based recombinant inbred lines of rice (*****Oryza sativa *****L.).** *BMC Plant Biol* 2012, **12:**137. 10.1186/1471-2229-12-137 PMC3438134 22876968

[11] Gao ZY, Zhao SC, He WM, Guo LB, Peng YL, Wang JJ, Guo XS, Zhang XM, Rao YC, Zhang C, Dong GJ, Zheng FY, Lu CX, Hu J, Zhou Q, Liu HJ, Wu HY, Xu J, Ni PX, Zeng DL, Liu DH, Tian P, Gong LH, Ye C, Zhang GH, Wang J, Tian FK, Xue DW, Liao Y, Zhu L (2013). Dissecting yield-associated loci in super hybrid rice by resequencing recombinant inbred lines and improving parental genome sequences. Proc Natl Acad Sci U S A.

[12] Xu P, Wu X, Wang B, Hu T, Lu Z, Liu Y, Qin D, Wang S, Li G: **QTL mapping and epistatic interaction analysis in asparagus bean for several characterized and novel horticulturally important traits.** *BMC Genet* 2013, **14:**4. 10.1186/1471-2156-14-4 PMC3616928 23375055

[13] Pfender WF, Saha MC, Johnson EA, Slabaugh MB (2011). Mapping with RAD (restriction-site associated DNA) markers to rapidly identify QTL for stem rust resistance in Lolium perenne. Theor Appl Genet.

[14] Chutimanitsakun Y, Nipper RW, Cuesta-Marcos A, Cistue L, Corey A, Filichkina T, Johnson EA, Hayes PM: **Construction and application for QTL analysis of a Restriction Site Associated DNA (RAD) linkage map in barley.** *BMC Genomics* 2011, **12:**4. 10.1186/1471-2164-12-4 PMC3023751 21205322

[15] Wei LB, Zhang HY, Zheng YZ, Miao HM, Zhang TZ, Guo WZ (2009). A Genetic Linkage Map Construction for Sesame (*Sesamum indicum* L.). Genes Genom.

[16] Zhang HY, Wei LB, Miao HM, Zhang TD, Wang CY: **Development and validation of genic-SSR markers in sesame by RNA-seq.** *BMC Genomics* 2012, **13:**316. 10.1186/1471-2164-13-316 PMC3428654 22800194

[17] Zhang H, Miao H, Wei L, Li C, Zhao R, Wang C: **Genetic analysis and QTL mapping of seed coat color in sesame (*****Sesamum indicum*****L.).** *PLoS One* 2013, **8**(5):e63898. 10.1371/journal.pone.0063898 PMC3660586 23704951

[18] Davey JW, Hohenlohe PA, Etter PD, Boone JQ, Catchen JM, Blaxter ML (2011). Genome-wide genetic marker discovery and genotyping using next-generation sequencing. Nat Rev Genet.

[19] Hyten DL, Cannon SB, Song Q, Weeks N, Fickus EW, Shoemaker RC, Specht JE, Farmer AD, May GD, Cregan PB: **High-throughput SNP discovery through deep resequencing of a reduced representation library to anchor and orient scaffolds in the soybean whole genome sequence.** *BMC Genomics* 2010, **11:**38. 10.1186/1471-2164-11-38 PMC2817691 20078886

[20] Chen S, Huang Z, Dai Y, Qin S, Gao Y, Zhang L, Chen J: **The development of 7E chromosome-specific molecular markers for*****Thinopyrum elongatum*****based on SLAF-seq technology.** *PLoS One* 2013, **8**(6):e65122. 10.1371/journal.pone.0065122 PMC3677899 23762296

[21] Sun X, Liu D, Zhang X, Li W, Liu H, Hong W, Jiang C, Guan N, Ma C, Zeng H, Xu C, Song J, Huang L, Wang C, Shi J, Wang R, Zheng X, Lu C, Wang X, Zheng H: **SLAF-seq: an efficient method of large-scale de novo SNP discovery and genotyping using high-throughput sequencing.** *PLoS One* 2013, **8**(3):e58700. 10.1371/journal.pone.0058700 PMC3602454 23527008

[22] Wang XQ, Zhao L, Eaton DA, Li DZ, Guo ZH (2013). Identification of SNP markers for inferring phylogeny in temperate bamboos (Poaceae: Bambusoideae) using RAD sequencing. Mol Ecol Resour.

[23] Barchi L, Lanteri S, Portis E, Acquadro A, Vale G, Toppino L, Rotino GL: **Identification of SNP and SSR markers in eggplant using RAD tag sequencing.** *BMC Genomics* 2011, **12:**304. 10.1186/1471-2164-12-304 PMC3128069 21663628

[24] Miller MR, Dunham JP, Amores A, Cresko WA, Johnson EA (2007). Rapid and cost-effective polymorphism identification and genotyping using restriction site associated DNA (RAD) markers. Genome Res.

[25] Hegarty M, Yadav R, Lee M, Armstead I, Sanderson R, Scollan N, Powell W, Skot L (2013). Genotyping by RAD sequencing enables mapping of fatty acid composition traits in perennial ryegrass (*Lolium perenne* L.). Plant Biotechnol J.

[26] Pegadaraju V, Nipper R, Hulke B, Qi L, Schultz Q: **De novo sequencing of sunflower genome for SNP discovery using RAD (Restriction site Associated DNA) approach.** *BMC Genomics* 2013, **14**(1):556. 10.1186/1471-2164-14-556 PMC3765701 23947483

[27] Wang N, Fang L, Xin H, Wang L, Li S: **Construction of a high-density genetic map for grape using next generation restriction-site associated DNA sequencing.** *BMC Plant Biol* 2012, **12:**148. 10.1186/1471-2229-12-148 PMC3528476 22908993

[28] Barchi L, Lanteri S, Portis E, Vale G, Volante A, Pulcini L, Ciriaci T, Acciarri N, Barbierato V, Toppino L, Rotino GL: **A RAD tag derived marker based eggplant linkage map and the location of QTLs determining anthocyanin pigmentation.** *PLoS One* 2012, **7**(8):e43740. 10.1371/journal.pone.0043740 PMC3422253 22912903

[29] Zhang Y, Wang L, Xin H, Li D, Ma C, Ding X, Hong W, Zhang X: **Construction of a high-density genetic map for sesame based on large scale marker development by specific length amplified fragment (SLAF) sequencing.** *BMC Plant Biol* 2013, **13**(1):141. 10.1186/1471-2229-13-141 PMC3852768 24060091

[30] Hua JP, Xing YZ, Xu CG, Sun XL, Yu SB, Zhang QF (2002). Genetic dissection of an elite rice hybrid revealed that heterozygotes are not always advantageous for performance. Genetics.

[31] Wu K, Yang M, Liu H, Tao Y, Mei J, Zhao Y: **Genetic analysis and molecular characterization of Chinese sesame (*****Sesamum indicum*****L.) cultivars using Insertion-Deletion (InDel) and Simple Sequence Repeat (SSR) markers.** *BMC Genet* 2014, **15**(1):35. 10.1186/1471-2156-15-35 PMC4234512 24641723

[32] Dixit A, Jin MH, Chung JW, Yu JW, Chung HK, Ma KH, Park YJ, Cho EG (2005). Development of polymorphic microsatellite markers in sesame (*Sesamum indicum* L.). Mol Ecol Notes.

[33] Cho YI, Park JH, Lee CW, Ra WH, Chung JW, Lee JR, Ma KH, Lee SY, Lee KS, Lee MC, Park YJ (2011). Evaluation of the genetic diversity and population structure of sesame (*Sesamum indicum* L.) using microsatellite markers. Genes Genom.

[34] Spandana B, Reddy VP, Prasanna GJ, Anuradha G, Sivaramakrishnan S (2012). Development and characterization of microsatellite markers (SSR) in Sesamum (*Sesamum indicum* L.) species. Appl Biochem Biotechnol.

[35] Yue WD, Wei LB, Zhang TD, Li C, Miao HM, Zhang HY (2012). Analysis of genetic diversity and population structure of germplasm resources in sesame (*Sesamum indicum* L.) by SSR markers. Acta Agronomica Sinica (Chinese).

[36] Wei W, Qi X, Wang L, Zhang Y, Hua W, Li D, Lv H, Zhang X: **Characterization of the sesame (Sesamum indicum L.) global transcriptome using Illumina paired-end sequencing and development of EST-SSR markers.** *BMC Genomics* 2011, **12:**451. 10.1186/1471-2164-12-451 PMC3184296 21929789

[37] Wang L, Zhang Y, Qi X, Gao Y, Zhang X (2012). Development and characterization of 59 polymorphic cDNA-SSR markers for the edible oil crop *Sesamum indicum* (Pedaliaceae). Am J Bot.

[38] Yepuri V, Surapaneni M, Kola V, Vemireddy LR, Jyothi B, Dineshkumar V, Anuradha G, Siddiq EA (2013). Assessment of genetic diversity in sesame (*Sesamum indicum* L.) genotypes, using EST-derived SSR markers. J Crop Sci Biotechnol.

[39] Xue S, Zhang Z, Lin F, Kong Z, Cao Y, Li C, Yi H, Mei M, Zhu H, Wu J, Xu H, Zhao D, Tian D, Zhang C, Ma Z (2008). A high-density intervarietal map of the wheat genome enriched with markers derived from expressed sequence tags. Theor Appl Genet.

[40] Xu P, Wu X, Wang B, Liu Y, Ehlers JD, Close TJ, Roberts PA, Diop NN, Qin D, Hu T, Lu Z, Li G: **A SNP and SSR based genetic map of asparagus bean (*****Vigna. unguiculata*****ssp.*****sesquipedialis*****) and comparison with the broader species.** *PLoS One* 2011, **6**(1):e15952. 10.1371/journal.pone.0015952 PMC3017092 21253606

[41] Brookes AJ (1999). The essence of SNPs. Gene.

[42] Lu H, Romero-Severson J, Bernardo R (2002). Chromosomal regions associated with segregation distortion in maize. Theor Appl Genet.

[43] Faris JD, Laddomada B, Gill BS (1998). Molecular mapping of segregation distortion loci in Aegilops tauschii. Genetics.

[44] Jia H, Wan H, Yang S, Zhang Z, Kong Z, Xue S, Zhang L, Ma Z (2013). Genetic dissection of yield-related traits in a recombinant inbred line population created using a key breeding parent in China’s wheat breeding. Theor Appl Genet.

[45] Palomeque L, Li-Jun L, Li W, Hedges B, Cober ER, Rajcan I (2009). QTL in mega-environments: I. Universal and specific seed yield QTL detected in a population derived from a cross of high-yielding adapted x high-yielding exotic soybean lines. Theor Appl Genet.

[46] Basunanda P, Radoev M, Ecke W, Friedt W, Becker HC, Snowdon RJ (2010). Comparative mapping of quantitative trait loci involved in heterosis for seedling and yield traits in oilseed rape (*Brassica napus* L.). Theor Appl Genet.

[47] Yang J, Zhu J, Williams RW (2007). Mapping the genetic architecture of complex traits in experimental populations. Bioinformatics.

[48] Liang HW, Wang CZ, Li Z, Luo XZ, Zou GW (2008). Improvement of the silver-stained technique of polyacrylamide gel electrophoresis. Yi Chuan.

[49] Baird NA, Etter PD, Atwood TS, Currey MC, Shiver AL, Lewis ZA, Selker EU, Cresko WA, Johnson EA: **Rapid SNP discovery and genetic mapping using sequenced RAD markers.** *PLoS One* 2008, **3**(10):e3376. 10.1371/journal.pone.0003376 PMC2557064 18852878

[50] Xu P, Xu S, Wu X, Tao Y, Wang B, Wang S, Qin D, Lu Z, Li G (2014). Population genomic analyses from low-coverage RAD-Seq data: a case study on the non-model cucurbit bottle gourd. Plant J.

[51] Catchen JM, Amores A, Hohenlohe P, Cresko W, Postlethwait JH (2011). Stacks: building and genotyping Loci de novo from short-read sequences. G3 (Bethesda).

[52] Paillard S, Schnurbusch T, Winzeler M, Messmer M, Sourdille P, Abderhalden O, Keller B, Schachermayr G (2003). An integrative genetic linkage map of winter wheat (*Triticum aestivum* L.). Theor Appl Genet.

[53] Van Os H, Stam P, Visser RG, Van Eck HJ (2005). RECORD: a novel method for ordering loci on a genetic linkage map. Theor Appl Genet.

[54] Voorrips RE (2002). MapChart: software for the graphical presentation of linkage maps and QTLs. J Hered.

[55] Schlotzhauer SD, Littell RC (1997). SAS System for Elementary Statistical Analysis.

[56] Wang S, Basten J, Zeng Z (2010). Windows QTL Cartographer 2.5.

[57] Yang J, Zhu J (2005). Methods for predicting superior genotypes under multiple environments based on QTL effects. Theor Appl Genet.

[58] Lander ES, Botstein D (1989). Mapping mendelian factors underlying quantitative traits using RFLP linkage maps. Genetics.

[59] Jiang C, Zeng ZB (1997). Mapping quantitative trait loci with dominant and missing markers in various crosses from two inbred lines. Genetica.

